# Neurorights in History: A Contemporary Review of José M. R. Delgado’s “Physical Control of the Mind” (1969) and Elliot S. Valenstein’s “Brain Control” (1973)

**DOI:** 10.3389/fnhum.2021.703308

**Published:** 2021-10-27

**Authors:** Stephan Schleim

**Affiliations:** Theory and History of Psychology, Heymans Institute for Psychological Research, Faculty of Behavioral and Social Sciences, University of Groningen, Groningen, Netherlands

**Keywords:** brain stimulation, brain reading, mind reading, brain-computer interface, neuroethics, neurolaw

## Abstract

Scholars from various disciplines discuss the ethical, legal, and social implications of neurotechnology. Some have proposed four concrete “neurorights”. This review presents the research of two pioneers in brain stimulation from the 1950s to 1970s, José M. R. Delgado and Elliot S. Valenstein, who also reflected upon the ethical, legal, and social aspects of their and other scientists’ related research. Delgado even formulated the vision “toward a psychocivilized society” where brain stimulation is used to control, in particular, citizens’ aggressive and violent behavior. Valenstein, by contrast, believed that the brain is not organized in such a way to allow the control or even removal of only negative processes without at the same time diminishing desirable ones. The paper also describes how animal and human experimentation on brain stimulation was carried out in that time period. It concludes with a contemporary perspective on the relevance of neurotechnology for neuroethics, neurolaw, and neurorights, including two recent examples for brain-computer interfaces.

## Introduction

Neurotechnology like neuroimaging, brain-computer interfaces, or brain stimulation raises important ethical, legal, and social questions. Different authors have identified four main rights to facilitate the discussion of such questions: the right to cognitive liberty, mental privacy, mental integrity, and psychological continuity (Bublitz, 2013; Ienca and Andorno, 2017; Ienca, 2021). I shall present the views of two authorities on brain stimulation from the 1950s to 1970s, José M. R. Delgado (1915–2011) and Elliot S. Valenstein (born 1923). Both scientists are remarkable in that they did not only apply this neurotechnology very early but also reflected on its potentials and risks for society; Delgado even imagined a whole society based on brain stimulation. As it will turn out, the views of both researchers are still relevant to the discussion of neurorights today. For this, the four neurorights mentioned before will be briefly summarized and compared to the historical period of brain stimulation. The following section addressing two contemporary examples of brain-computer interfaces will emphasize the importance of precisely understanding the psychological concepts and processes involved in such experiments. I shall conclude with a brief outlook on aspects deserving special consideration in future ethical and legal assessments of neurotechnology, particularly with respect to neurorights.

## Delgado and The “Psychocivilized Society”

José M. R. Delgado was born in Spain, received a Ph.D. from the Ramón y Cajal Institute in Madrid, and accepted a position in the physiology department of Yale University under John F. Fulton (1899–1960), a pioneer in neurosurgery (Fulton, 1949, 1951), in 1950.^1^ He had already authored papers on brain stimulation in animals in 1952 (Delgado, 1952a,b) and later published repeatedly in *Science* (Delgado, 1963, 1965). In 1969, he published the popular science book “Physical Control of the Mind: Toward a Psychocivilized Society” in which he explains brain stimulation and which has 831 Google Scholar citations.^2^ Only a year later, the *New York Times* published a long interview with him, titled “Brain Researcher José Delgado Asks—‘What kind of Humans Would We Like to Construct?’ ”^3^ Years after his death, he is remembered as “The Man Who Fought A Bull With Mind Control”^4^.

Delgado’s book starts out with general parts on human evolution (Part I) and the brain (Part II). He then describes brain stimulation and its experimental application, mostly in cats, monkeys, and humans (Part III), followed by a theoretical evaluation (Part IV). The book concludes with his vision “Toward a Psychocivilized Society” (Part V). Delgado describes how they first implanted electrodes in animals’ brains and then, when this proved safe enough, extended the method to human subjects for diagnostic and therapeutic purposes.^5^ His devices did not only allow electrical stimulation but also recorded electrical signals and were remotely controlled: “[I]t is already possible to equip animals or human beings with minute instruments called ‘stimoceivers’ for radio transmission and reception of electrical messages to and from the brain in completely unrestrained subjects” (Delgado, 1971: 90). At length, he explains:

“*Leaving wires inside of a thinking brain may appear unpleasant or dangerous, but actually the many patients who have undergone this experience have not been concerned about the fact of being wired, nor have they felt any discomfort due to the presence of conductors in their heads. Some women have shown their feminine adaptability to circumstances by wearing attractive hats or wigs to conceal their electrical headgear, and many people have been able to enjoy a normal life as outpatients, returning to the clinic periodically for examination and stimulation. In a few cases in which contacts were located in pleasurable areas, patients have had the opportunity to stimulate their own brains by pressing the button of a portable instrument, and this procedure is reported to have therapeutic benefits.”* (Delgado, 1971: 88).

In cats and monkeys, electrodes were placed in the amygdala or hypothalamus, for example, and brain stimulation was used to trigger or suppress aggressive behavior. By putting these animals together in cages with others, Delgado investigated whether the social relations and hierarchies could be changed by evoking (or suppressing) such behavior. He continues to discuss attempts to induce emotions of anxiety, fear, pleasure, or violent behavior in patients and convicted criminals. A “typical example” is J. P., “a charming and attractive 20-year-old girl with a history of encephalitis at the age of 18 months and many crises of temporal lobe seizures and grand mal attacks for the last 10 years” (Delgado, 1971: 132). That woman had regular outbursts of rage and had already attacked two persons with a knife or a pair of scissors. Delgado writes about the implantation of the electrodes and the experimentation:

“*The patient was committed to a ward for the criminally insane, and electrodes were implanted in her amygdala and hippocampus for exploration of possible neurological abnormalities. As she was rather impulsive, confinement in the EEG recording room was impractical, and she became one of the first clinical cases instrumented with a stimoceiver, which made it possible to study intracerebral activity without restraint. […] During depth explorations, it was demonstrated that crises of assaultive behavior similar to the patient’s spontaneous bursts of anger could be elicited by radio stimulation of contact 3 in the right amygdala. A 1.2 milliampere excitation of this point was applied while she was playing the guitar and singing with enthusiasm and skill. At the 7th second of stimulation, she threw away the guitar and in a fit of rage launched an attack against the wall and then paced around the floor for several minutes, after which she gradually quieted down and resumed her usual cheerful behavior. This effect was repeated on two different days.”* (Delgado, 1971: 137).

In another patient, stimulation of the amygdala could, by contrast, *diminish* aggressive behavior (Delgado et al., 1968). Delgado also investigated the induction of hallucinations and memories in humans. It is sometimes reported in the secondary literature that electrical brain stimulation can give rise to illusions of the will (for a review, see Selimbeyoglu and Parvizi, 2010; Schleim, 2012a). But according to Delgado’s reports the patients either knew that a sensation/behavior was triggered externally^6^ or the situation was unclear. The observation of the change of the social hierarchy among monkeys is likely to have inspired his social vision, to which I will turn after this remarkable quote about the leader in such a colony who has become more peaceful through brain stimulation:

“*This social dominance has been abolished by stimulation applied for 5 seconds once a minute for 1 hour to the caudate nucleus in the boss monkey. During this period the animal’s facial expression appeared more peaceful both to the investigator and to the other animals, who started to circulate freely around the cage without observing their usual respect. […] The old dream of an individual overpowering the strength of a dictator by remote control has been fulfilled, at least in our monkey colonies, by a combination of neurosurgery and electronics, demonstrating the possibility of intraspecies instrumental manipulation of hierarchical organization.”* (Delgado, 1971: 164–166).

In the chapter titled “Ethical Considerations” towards the end of the book, he summarizes:

“*The individual is defenseless against direct manipulation of the brain because he is deprived of his most intimate mechanisms of biological reactivity. In experiments, electrical stimulation of appropriate intensity always prevailed over free will; and, for example, flexion of the hand evoked by stimulation of the motor cortex cannot be voluntarily avoided. Destruction of the frontal lobes produced changes in affectiveness which are beyond any personal control.”* (Delgado, 1971: 214).

Delgado compares neurotechnology to a knife, “neither good nor bad; but it may be used by either a surgeon or an assassin” (Delgado, 1971: 215). He then contrasts it with neurosurgery and quotes an extreme case where a serial robber had undergone frontal lobe surgery as an alternative for a long jail sentence. After initial improvement, the criminal behavior would have reappeared several months later: “When he realized that the police were closing in, he wrote a letter to the surgeon expressing appreciation for his efforts and regret that the operation had not been successful. Hoping that the study of his case might help others, he donated his brain to the surgeon and committed suicide by shooting himself through the heart” (Delgado, 1971: 220). Society would already limit individual freedom in other cases, for example, requiring a negative syphilis test before allowing a couple to marry. But, thanks to his research and that of others: “We are now on the verge of a process of mental liberation and self-domination which is a continuation of our evolution” (Delgado, 1971: 223). A few pages later, he starts describing his vision of a “psychocivilized society”, comparing the breakthroughs of brain research with the Copernican and Darwinian revolution in science, similar to how Sigmund Freud did when he developed psychoanalysis (Freud, 1917/1955). In Delgado’s words: “We may now be approaching a third equally momentous discovery about ourselves. The analysis of mental activities in the context of brain physiology indicates that our own self, our ego, is not so unique or even independent, as Freud pointed out many years ago” (Delgado, 1971: 232). Brain stimulation could help overcome limitations due to our genes or the environment in which we grew up. People could also be manipulated through ill-meaning educators.

He continues to develop a program of “psychogenesis”, stating that “[m]an is not born free but subservient to genes and education” and that “[p]ersonal freedom is not inherited nor is it a gift of nature, but one of the highest attainments of civilization” which requires us to “choose consciously and intelligently among environmental alternatives” (Delgado, 1971: 242). He then discusses the discovery of atomic energy, the development of the nuclear bomb, and well-known novels like Huxley’s “Brave New World”, Orwell’s “1984”, and Condon’s “The Manchurian Candidate”. Society was facing the threats of destruction, alienation, and environmental pollution; progress had not increased happiness. He writes that many such problems were due to the fact that our material evolution is going quicker than our mental evolution:

“*We are civilized in our physical ecological accomplishments but barbaric in our psychological responses. Within some limits, we can control atoms, trees, and animals, while we have not learned to control ourselves. New solutions are needed in order to civilize our psyche, consciously to organize our efforts to develop a future psychocivilized society.”* (Delgado, 1971: 254).

He concludes his book with a tentative plan consisting of five steps: First, more scientific investigation, promoted and funded by the government which should declare “conquering of the human mind’ a national goal at parity with conquering of poverty or landing a man on the moon” (Delgado, 1971: 259). Second, interdisciplinary communication, combining biology and the neural sciences with sociology, pedagogy, and philosophy.^7^ Third, a manpower shift making more people work in the neurobehavioral field. Fourth, better (in particular psychological) education of the youth. And fifth, better education of the general public about the brain, mind, and behavior.

Note that the intention of this section was to describe José Delgado’s views neutrally, without discussion or evaluation. Valenstein’s different views in the next section will already put Delgado’s psychocivilized society in perspective, before I relate this research to contemporary views on neurorights.

## Valenstein’S “Brain Control”

Elliot S. Valenstein was born in the United States and is a Professor Emeritus of Psychology and Neuroscience at the University of Michigan. He received his Ph.D. from the University of Kansas and held different research positions before joining the University of Michigan in 1970.^8^ Like Delgado, he repeatedly published on brain stimulation in *Science* (Valenstein and Beer, 1962; Valenstein and Valenstein, 1964; Valenstein et al., 1968). His book “Brain Control: A Critical Examination of Brain Stimulation and Psychosurgery” also addresses a broad, but arguably more educated readership (Valenstein, 1973). It’s more comprehensive with its 407 pages than Delgado’s 280 and Valenstein doesn’t develop a social vision but rather informs his readers about facts and myths on brain control. The book’s first part summarizes the history and experimental evidence; the second part describes clinical and social applications and concludes with a discussion of ethical and social aspects. The book has 446 Google Scholar citations. In its bibliography, José Delgado is the most frequently mentioned first author (with 14 publications), without Valenstein himself (16 publications).

Valenstein discusses whether brain stimulation could produce a population of slaves or robots and concludes: “Brain stimulation technology should be examined in other contexts besides those related to the very remote possibility that it can be used to control individuals or groups of people” (Valenstein, 1973: 85). There were a great number of reports, though, suggesting the possibility to manipulate motivation and emotion in very predictable ways, which made the technology a possible solution to medical and social problems. He describes and discusses illustrative experiments, including Delgado’s famous bullfight where the neuroscientist stopped an attacking bull with an implanted stimoceiver. Valenstein suggests that in contrast to Delgado’s interpretation and popular accounts in the media, the stimulation in the bull’s caudate nucleus would probably have interrupted motor behavior more generally instead of making the animal specifically less aggressive. After a summary of experimentation with human subjects, Valenstein describes five limitations of brain stimulation: First, the limited precision with which the electrodes can be placed; second, inter-individual brain variability; third, the way individual personality and history shape responses to stimuli; fourth, the situational dependency of responses (we might now say “situatedness”); and fifth, diachronic variability due to learning (what we would now call “plasticity”). Valenstein then discusses in much detail the potential and risks of brain stimulation and psychosurgery in regulating aggression, violence, and sexuality in medical and criminal contexts. Psychosurgery deserves a discussion on its own and brain stimulation has been addressed in more detail above. I shall thus limit the remainder of this section to Valenstein’s ethical and social considerations.

The central notion in his discussion is informed consent—but also coercion, which is particularly salient in criminal contexts where brain stimulation or surgery could be offered as an alternative to punishment. Valenstein makes four concrete recommendations for Ethical Review Boards: First, the members should be as independent as possible from doctors or researchers carrying out the procedure; second, alternatives should be considered and an ombudsman should be involved to represent the patient’s perspective, particularly for children; third, there should be a clear rationale for the proposed procedure; and fourth, when patients are involved there should be honesty on whether they directly benefit from the procedure or are rather used for experimental purposes: “There is little doubt that electrodes have been inserted in diverse regions of the brain where the probability of obtaining information of direct benefit to the patient has to be regarded as extremely remote” (Valenstein, 1973: 341). In a couple of cases, such stimulation would have led to strong emotional responses with serious psychological implications.

Considering society at large, Valenstein primarily addresses the problem of crime and quotes from the Presidential Address by social psychologist Kenneth B. Clark at the American Psychological Association (APA) gathering in 1971:

“*Given the urgency of the immediate survival problem, the psychological and social sciences must enable us to control the animalistic, barbaric and primitive propensities in man and subordinate these negatives to the uniquely human moral and ethical characteristics of love, kindness, and empathy. […] We can no longer afford to rely solely on the traditional prescientific attempts to contain human cruelty and destructiveness.”* (Clark, as quoted by Valenstein, 1973: 350).

Clark later refers to “psychotechnological, biochemical intervention” as a possible solution, but Valenstein accuses him of representing a “modern phrenology” because the brain is not organized in such a way that negative aggression could be suppressed specifically without diminishing desirable capacities as well. He concludes his book, stating:

“*[T]here is a great danger in accepting the delusion that biological solutions are available for these social problems. It is likely that there are some biological factors that contribute to a propensity toward violence, but we would be in serious trouble if a number of influential people became convinced that violence is mainly a product of a diseased brain rather than a diseased society.”* (Valenstein, 1973: 353).

## Brain Stimulation and Contemporary Neurorights

This section discusses whether the brain stimulation research of the 1950s to 1970s as well as its ethical reflection is still relevant to the present discussion of neurorights (e.g., Bublitz, 2013; Ienca and Andorno, 2017; Ienca, 2021), neuroethics, and neurolaw (Schleim, 2012b,c; Schleim, 2020; Muñoz et al., 2020; Ligthart et al., 2021). For this, I will briefly summarize the main neurorights addressed in the literature and compare them to the historical research on brain stimulation described above. It should be noted that these rights are related to further ethical and legal concepts and traditions, whose discussion goes beyond the scope of this review. The reader interested in these aspects should study the primary publications on neurorights.

As already stated in the introduction, the four main neurorights are: (1) cognitive liberty; (2) mental privacy; (3) mental integrity; and (4) psychological continuity. The first, sometimes also called the right to mental self-determination, has two aspects: access to neurotechnologies and protection against their coercive and unconsented use; cognitive liberty is also considered the most fundamental one, giving an individual the right and freedom to determine their own mental processes. The second emphasizes the personal and sensible nature of brain data, similar to personal data users create and leave on information processing systems; it goes without saying that where already behavioral data fall under the right to privacy, data recorded from within people’s skulls are potentially even more sensible, as they might give away information someone wants to hide in their behavior in certain contexts, such as the health state, sexual preference, or political views. The third is sometimes discussed in analogy to hacking a computer: someone might abuse a brain-computer interface to change a person’s psychological processes (Ienca and Haselager, 2016). The fourth is about people’s perception of their own identity in the course of time; the neuroright to psychological continuity could be violated when neurotechnology is used in such a way that someone’s personality or personal identity is changed. Note that the literature on neurorights also addresses the question whether these rights should be understood in an *absolute* manner, such that no restriction or violation of them would be justified, or whether they are *relative* in the sense that an individual’s consent or the protection of other people’s rights—such as the right to life by preventing a serious crime—might justify their restriction or violation.

Delgado and Valenstein both did their research in the Cold War period when scenarios like brain control apparently seemed very realistic, to which the former contributed himself with his vision of the psychocivilized society, but which the latter described as unlikely.^9^ The idea that human thought and behavior must be controlled to prevent disaster seems to have been particularly prevalent then, as also expressed by the former APA President Kenneth B. Clark. While Valenstein expressed a critical stance towards brain stimulation, actually proposing ethical considerations for clinical research himself and discussing limitations of the technology’s possible application, Delgado even saw his advanced stimoceiver as a neutral tool to be used for the better or worse of individuals and humankind. Even stronger, the latter firmly believed that the future of humanity depended on the possibility to control people’s minds through controlling their brains. He assumed that the clinical applications justified the development of brain reading and stimulation devices anyway and that as soon as they worked reliably, they would be adopted by society at large^10^.

Brain stimulation as discussed by Valenstein obviously touches on fewer neurorights as Delgado’s stimoceiver, which combines a brain recording and stimulation device. The first neuroright, cognitive liberty, is relevant to both technologies: People might demand access to the means to change their psychological processes in a desired way and they need to be protected from their coercive and involuntary application. The second, mental privacy, is particularly relevant to recording brain data, as with Delgado’s device; it is likely, though, that the application of brain stimulation presumes another neurotechnology, such as neuroimaging, to identify a suitable target for intervention, which would then require the recording of sensible brain data. By contrast, the third and fourth, mental integrity and psychological continuity, are particularly relevant to neurotechnology changing brain processes, such as brain stimulation; but this again might presume some recording of brain data to adjust the technology to a particular individual.

This brief discussion demonstrates two points: first, the neurodevices developed and applied already in the 1950s to 1970s can be evaluated from the perspective of contemporary neurorights. They are thus an interesting precursor to the present technology and should not be forgotten (Hariz et al., 2010). Second, while the particular features of neurotechnology—such as brain recording as opposed to stimulation/intervention capabilities—have different implications for the four neurorights, it might be that in clinical practice or everyday applications all neurorights are involved. This is likely due to this review’s focus on brain stimulation, which also presumes some knowledge about the brain to be stimulated. It goes without saying that concepts like personal identity, which was central for the fourth neuroright, are very complex and deserve a much deeper discussion, which has been provided elsewhere (e.g., Merkel et al., 2007). However, before concluding with a summary and outlook, I would like to briefly address recent examples of brain-computer interfaces to illustrate the importance of a proper conceptual analysis for the assessment of neurotechnology from a neurorights perspective.

## Brain-Computer Interfaces in The 21st Century

This review thus far has shown that historical precursors of contemporary neurotechnology can be discussed meaningfully from the perspective of neurorights. But ethical and legal evaluation should ideally allow us to guide present and future applications of such and similar devices. Such an assessment, in my view, presumes a proper understanding of a technology’s possibilities and limitations. Delgado and Valenstein were both pioneers in the field of brain stimulation. Yet, as we have seen above, they expressed very different perspectives on that technology’s potential to a broader public. Delgado’s vision of a psychocivilized society might remind some readers of exaggerations in contemporary media and the science fiction literature. With two recent examples of scientific brain reading as enabled by brain-computer interfaces I want to demonstrate the importance of a proper conceptual analysis of a neurotechnology’s possibilities and limitations, which is particularly relevant to mental privacy.

Notwithstanding the progress in psychology and neuroscience of the last decades, much about the human mind and consciousness remains a riddle. For example, researchers may predict that electrical stimulation in the temporal lobe leads to auditory or visual hallucinations, but not what their precise *content* will be (Selimbeyoglu and Parvizi, 2010). Guillory and Bujarski reviewed the emotional responses associated with intracranial brain stimulation during 60 years of research (Guillory and Bujarski, 2014). Amygdala stimulation, for example, was primarily associated with fear, but also with happiness, anger, and sadness. Happiness, the other way around, also occurred when the anterior cingulate gyrus, the supplementary motor area, the inferior frontal gyrus, or other areas were stimulated. And this still neglects the conceptual point that there is no single, generally accepted clear definition of notions like “emotion” or “happiness”. It was precisely this incapacity of “introspective psychology” (Danziger, 1980) to provide clear definitions of such mental vocabulary that fueled John B. Watson’s (1878–1958) influential behavioristic paradigm (Watson, 1913) and made Burrhus F. Skinner (1904–1990), another leading behaviorist, skeptical of mental terms in general (Skinner, 1971).

This *conceptual* point and the “mental ontology” researchers are using when probing their subjects’ brains becomes relevant when translating brain signals into psychological terms, as can be illustrated with the following two examples: A few years ago, researchers built a brain-computer interface enabling, in their words, “conscious brain-to-brain communication in humans using non-invasive technologies” (Grau et al., 2014). Some media described this as a form of “telepathy” and “mind control”.^11^ The researchers had translated the Spanish and Italian words for “hello” and “bye” (“hola” and “ciao”) into a binary code and then associated voluntary motor imagery of the *hands* with the value 1 and of the *feet* with the value 0. The associated neural patterns could be identified from EEG signals from the *transmitting* subjects. The *receivers* were connected to a transcranial magnetic (TMS) coil targeting their visual cortex and should report, at a pre-defined moment, whether they could see a light flash, which was defined as 1, and otherwise 0. It is important to understand the researchers’ conventions on which such brain reading systems are based when discussing their impact on neurorights. The subjects imagined limb movements or saw a light flash, but the particular meaning (i.e., “hello” or “bye”) was given to the associated brain signals only in that particular experimental context.

In a more sophisticated and clinical setting, another group of researchers used a brain-computer interface to allow a woman in an advanced locked-in state communication with the outer world (Vansteensel et al., 2016). Guided by neuroimaging, neurosurgery was used to place electrodes directly on the patient’s cortical brain tissue, allowing a better recording quality than an external electroencephalography (EEG) device. These electrodes were connected to an implanted device in the chest, which in turn could communicate with an external receiver linked to a tablet computer. The woman then exercised certain computer tasks throughout a period of 38 weeks after implantation, to allow the researchers to calibrate the pattern recognition algorithm. That brain-computer interface allowed the locked-in patient to spell words at the rate of initially 52 s per character, which eventually could be reduced to 33 s per character when using word prediction. The cognitive task most suitable for the system was the attempt to move the right hand for approximately 1 s, which the computer interpreted as a trigger to select the presently highlighted character. The electrodes utilized for this task were those on the patient’s left sensorimotor area associated with right-hand movements.

It goes without saying that such or similar systems can have a major impact for patients otherwise unable to communicate or trying to control a prosthetic limb. However, from the perspective of neurorights and in particular mental privacy, a closer analysis reveals that the signals recorded here are not very sensible. This does not rule out the possibility that other or future approaches might record much more personal and private data but emphasizes once more that a proper understanding of a technology’s possibilities and limitations is a prerequisite for a meaningful ethical and legal assessment, also from the perspective of neurorights. In the final section, I will close with some suggestions on which aspects should be considered for such assessments in the future.

## Summary and Outlook

This review illustrated the relevance of historical research on brain stimulation to the contemporary discussion of neurorights. The early research of José M. R. Delgado and Elliot S. Valenstein was remarkable not only because of its technical refinement but also due to the researchers’ social and ethical perspectives, described in their books and addressed at broader audiences. We now know that the former was too optimistic and that the latter’s perspective remains more valid until today. This applies in particular to Valenstein’s description of inter-individual brain differences, the brain’s plasticity, and its situatedness, limiting the possibilities of brain control.

There are many other ways of recording data or intervening in the brain which could not be addressed here, such as psychosurgery, psychopharmacology, or brain imaging and electrical stimulation devices already available for consumers at large. One central aspect, on my account, for the ethical and legal assessment will be whether the *psychological* *meaning* of recorded signals can be derived from the brain alone or has to be interpreted by experimenters, as was the case for the brain-computer interfaces described in the previous section. This added layer of interpretation makes present neurotechnology seem less problematic from the perspective of neurorights. Similarly, Valenstein’s suggestion that Delgado’s famous bull experiment rather blocked the animal’s motor system than controlling its consciousness, is important for a proper ethical and legal assessment. The discussion on mental privacy or personal continuity, by contrast, will also essentially depend on how central notions like privacy or personal identity are understood. This illustrates that neuroethics, neurolaw, and neurorights are truly interdisciplinary fields.

## Data Availability Statement

The original contributions presented in the study are included in the article material, further inquiries can be directed to the corresponding author.

## Author Contributions

SS conceived and wrote the whole manuscript.

## Conflict of Interest

The author declares that the research was conducted in the absence of any commercial or financial relationships that could be construed as a potential conflict of interest.

## Publisher’s Note

All claims expressed in this article are solely those of the authors and do not necessarily represent those of their affiliated organizations, or those of the publisher, the editors and the reviewers. Any product that may be evaluated in this article, or claim that may be made by its manufacturer, is not guaranteed or endorsed by the publisher.

## Footnotes

^1^Please note that I focus on the researchers’ views on neurotechnology and their relevance to the present discussion of neurorights in this paper. I shall therefore be very brief on the biographical details.

^2^Partially based on the Salmon Lectures (named after Thomas W. Salmon, MD) at the New York Academy of Sciences in December 1968 and written during a sabbatical under a Guggenheim Fellowship. I shall quote from the first Harper & Row edition of 1971 that I own (Delgado, 1971).

^3^November 15, 1970, online at https://www.nytimes.com/1970/11/15/archives/brain-researcher-jose-delgado-asks-what-kind-of-humans-would-we.html (accessed April 29, 2021)

^4^Discover Magazine, May 21, 2016, online at and including an original video of the bullfight: https://www.discovermagazine.com/mind/the-man-who-fought-a-bull-with-mind-control (accessed April 29, 2021). See also Snyder (2009) for a discussion of the media’s role in this case.

^5^A scientific account of the application in patients can be found in Delgado et al. (1968).

^6^E.g., “I guess, Doctor, that your electricity is stronger than my will” (Delgado, 1971: 114).

^7^He probably does not mention psychology here, as he addresses disciplines having less contact with the neural sciences.

^8^See his short biography on the university website at: https://lsa.umich.edu/psych/people/emeriti-faculty/esv.html (accessed April 30, 2021)

^9^One might describe Valenstein’s view as an example of Engel’s biopsychosocial model (Engel, 1977), particularly when he writes about criminality. Note that Adrian Raine, a present leader in neurocriminology, could probably also be described as taking that stance, looking further than the one-sided title of his “The Anatomy of Violence: The Biological Roots of Crime” (Raine, 2014; see also Schirmann and Schleim, 2014).

^10^Vera and Martínez-Sánchez (2016) suggest that the measurable and statistically significant decrease in references to Delgado’s research might be due to increasing ethical concerns about his research.

^11^For example, Yale Scientific of November 4, 2015, online at: https://www.yalescientific.org/2015/11/science-or-science-fiction-telepathy-and-mind-control/ (accessed April 30, 2021)
